# Characterization and spinal fusion effect of rabbit mesenchymal stem cells

**DOI:** 10.1186/1756-0500-6-528

**Published:** 2013-12-10

**Authors:** Tsung-Han Lee, Yu-Hua Huang, Nyuk-Kong Chang, Wan-Ching Lin, Pei-Wen Chang Chien, Tsung-Ming Su, Dar-Jen Hsieh, Tao-Chen Lee

**Affiliations:** 1 Graduate Institute of Medicine, College of Medicine, Kaohsiung Medical University, Kaohsiung, Taiwan; 2 Department of Neurosurgery, Kaohsiung Chang Gung Memorial Hospital and Chang, Gung University College of Medicine, Kaohsiung, Taiwan; 3 Department of Center for Laboratory Animals, Kaohsiung Chang Gung Memorial, Hospital and Chang Gung University College of Medicine, Kaohsiung, Taiwan; 4 Sunmax Biotechnology CO., LTD., Tainan, Taiwan; 5 Department of Neurosurgery, Chiayi Chang Gung Memorial Hospital, Chiayi, Taiwan

**Keywords:** Characterization, Spinal fusion, Mesenchymal stem cell

## Abstract

**Background:**

The surface markers of mesenchymal stem cells (MSCs) of rabbits have been reported only sporadically. However, interest in the spinal fusion effect of MSCs has risen recently. The purpose of this research was to study the surface markers and spinal fusion effect of rabbit MSCs.

**Results:**

Of our rabbit MSCs, 2% expressed CD14, CD29, and CD45, 1% expressed CD90 and 97% expressed CD44. These results implied the MSCs were negative for CD14, CD29, CD45, and CD90, but positive for CD44. The surgical results showed that satisfactory fusion occurred in 10 rabbits (83%) in the study group and unsatisfactory fusion in 2 (17%). In the control group, satisfactory fusion was found in 3 rabbits (25%) and unsatisfactory fusion in 9 (75%). Statistical analysis showed the study group had significantly better spinal fusion results than the control group.

**Conclusions:**

The surface markers of human and rabbit MSCs are not exactly the same. Rabbit MSCs do not have positive reactivity for CD29 and CD90, which are invariably present on human MSCs. The allogeneic undifferentiated rabbit MSCs were able to promote spinal fusion and did not induce an adverse immune response.

## Background

Biological and clinical interest in mesenchymal stem cells (MSCs) has risen dramatically over the past two decades [1,2]. MSCs are multipotent cells that can replicate and have the potential to differentiate to lineages of mesenchymal tissues, including bone, cartilage and fat [2]. MSCs can be isolated from bone marrow and expanded in cultures. Adult bone marrow contains a heterogeneous population of cells, including hematopoietic stem cells, macrophages, erythrocytes, fibroblasts, adipocytes, and endothelial cells. In addition to these cell types, bone marrow also contains a subset of nonhematopoietic stem cells that possess a multilineage potential [3,4]. These properties make bone marrow-derived MSCs a good candidate for potential therapeutic applications such as cellular and gene therapies, tissue engineering and other preclinical investigations [5]. Human MSCs are characterized by the presence of a consistent set of marker proteins on their surface, including CD29, CD44, CD71, CD90, CD105, and an absence of marker proteins of hematopoietic lineage and leukocytes, including CD14, CD34, CD45 [2]. However, sporadic reports have suggested that the MSC surface markers of humans and rabbits may not be exactly the same [6,7].

The aim of the current study was to investigate the surface markers of rabbit MSCs using rabbit-specific antibodies and flow cytometry, and to determine whether allogeneic undifferentiated MSCs have the same good fusion effect as autologous differentiated MSCs, which have been advocated recently by other investigators [8].

## Methods

### Isolation and culture of rabbit bone marrow MSCs

The femurs of rabbits were harvested under general anesthesia and sterile conditions. Muscle and all connective tissue were detached from the femurs. The ends of the bones were cut away and an 18-gauge needle was inserted into the femoral shafts. The bone marrow of the shafts was extruded by flushing with low-glucose Dulbecco's Modified Eagle Medium (DMEM-LG; Gibco-BRL, Carlsbad, CA) supplemented with 10% fetal bovine serum (FBS) (Gibco-BRL, Carlsbad, CA), 100 U/ml penicillin (Hyclone, Logan, UT) and 100 μg/ml streptomycin (Hyclone, Logan, UT). Marrow plug suspension was dispersed by pipetting, filtered through a 70-μm mesh nylon filter (Becton Dickinson Biosciences, Bedford, MA), and centrifuged at 400 × g for 5 minutes. The pellet was resuspended in the RBC lysis buffer (0.154 M NH_4_Cl, 10 mM KHCO_3_ and 0.1 mM EDTA) (Panreac, Barcelona, Spain) for 5 minutes to lyse the red blood cells and centrifuged at 400 × g for 5 minutes. The supernatant was decanted by pipetting. Cells of 1 × 10^7^ were seeded in tissue culture plate (100 mm diameter) and incubated at 37°C in 5% CO_2_. After 4 days of incubation, the non-adherent cells were removed by replacing the medium. Thereafter, the medium was changed twice a week. At 80-90% confluence, cells were harvested with 0.05% trypsin-EDTA (Gibco, Carlsbad, CA) for 5-10 minutes at 37°C. The cells were centrifuged at 400 × g for 5 minutes. The resuspended cells were replated at 1.5 × 10^6^ cells per plate. The culture medium was changed twice a week.

We also prepared HIG-82 cells (BCRC 60242), rabbit synovial fibroblasts, which served as a control while we studied the differentiation potentials of our rabbit marrow cells. The HIG-82 cells were obtained from the Bioresource Collection and Research Center (BCRC, Hsinchu, Taiwan) and were cultured using the same methods as described above.

### Observation of differentiation potentials of rabbit MSCs

The rabbit bone marrow cells obtained from passage 3 were tested for the potential of differentiation into mesenchymal tissues.

For osteogenic differentiation, the rabbit cells were cultured for 3 weeks in DMEM-LG containing 10% FBS, 50 μg/ml ascorbic acid (Sigma-Aldich, St Louis, MO), 10 mM β-glycerophosphate (Calbiochem, San Diego, CA) and 10^-7^ M dexamathone (Sigma-Aldich, St Louis, MO). Then, the cells were rinsed with phosphate-buffered saline (PBS) (8 mM Na_2_HPO_4_, 150 mM NaCl, 2 mM KH_2_PO_4_, 3 mM KCl) (pH 7.4) (Sigma-Aldich, St Louis, MO) and fixed with 4% formaldehyde (Shimakyu Co., Ltd., Osaka, Japan) in PBS (pH 7.4) at room temperature for 10 minutes. Finally, the cells were incubated with 2% Alizarin Red (Sigma-Aldrich, St Louis, MO), pH 4.2, at room temperature for 30 minutes.

For chondrogenic differentiation, the rabbit cells were plated at 2.5 × 10^5^ cells/35 mm dish (6 well plates), cultured in chondrogenic medium for 3 weeks and stained with Alcian blue. We also used a 3D culture system [9]. Here is the protocol: 2.5 × 10^5^ rabbit MSCs were placed in a 15 ml polypropylene tube (Falcon, Bedford, MA), and centrifuged into pellets. The pellets were cultured at 37°C with 5% CO2 in 1 ml chondrogenic medium; half of the medium was exchanged for fresh medium every 2 to 3 days, for 3 weeks. Under microscopy, the pellets were embedded in paraffin, cut into 4 μm sections and stained with Alcian blue (Sigma-Aldrich, St Louis, MO) solution for 30 minutes at room temperature.

For adipogenic differentiation, the rabbit cells were cultured for 3 weeks in DMEM-LG containing 10% FBS, 0.5 mM isobutyl-methylxanthine (Sigma-Aldrich, St Louis, MO), 0.2 mM indomethacin (Sigma-Aldrich, St Louis, MO), 1 μM dexamethasone (Sigma-Aldrich, St Louis, MO) and 10 μg/mL insulin (Sigma-Aldrich, St Louis, MO). Then, the cells were rinsed with PBS (pH 7.4) and fixed with 4% formaldehyde in PBS (pH 7.4) at room temperature for 10 minutes. Finally, the cells were incubated with 0.5% Oil Red O (Sigma-Aldrich, St Louis, MO) solution for 30 minutes at room temperature.

As for the control, the cloned HIG-82 cells (rabbit fibroblasts) underwent the same staining methods with Alizarin Red, Alcian blue and Oil Red O, as described above. The staining results were observed using an inverted microscope.

### FACS analysis and flow cytometry of rabbit MSCs

The rabbit MSCs were characterized for the expression of surface markers of MSC using FACS analysis with anti-rabbit CD14 (Antigenix America, Huntington Station, NY), CD29 (Abcam, Cambridge, MA), CD44 (Antigenix America, Huntington Station, NY), CD45 (Antigenix America, Huntington Station, NY) and CD90 (Thermo Fisher Scientific, Waltham, MA) monoclonal antibodies. Briefly, the rabbit MSCs at passage 3 were harvested and suspended in their own culture medium at a concentration of 1 × 10^6^ cells/ml. The cells underwent primary staining with mouse anti-rabbit CD14, CD44, CD45, CD29 and CD90 monoclonal antibodies at room temperature for 30 minutes [2 × 10^5^ cells/0.1 ml in PBS/0.1% bovine serum albumin (BSA) (Sigma-Aldich, St Louis, MO)]. Then, the cells were stained with secondary antibody at room temperature for 30 minutes. The secondary antibody was rat anti-mouse IgG (H + L)-fluorescein isothiocyanate (FITC) (eBioscience, San Diego, CA). The cells labeled with surface antibodies were analyzed 4 times with flow cytometry on a FACS Caliber cytometer (Becton Dickinson Biosciences, Heidelberg, Germany). Prior to flow cytometric analysis, cell samples were washed and dead cells with debris were excluded. The data were analyzed with Cell Quest software (Becton Dickinson Biosciences, Heidelberg, Germany) and the percentage of marker-positive cells was shown by Mean ± SD (n = 4).

### Spinal fusion model

We have had extensive experience in performing Boden’s 1995 spinal fusion model [10-12]. Using this model [13], a bilateral posterolateral intertransverse-process fusion at the L5-6 level was performed in 24 male New Zealand white rabbits, each weighing about 3.0 kg and aged 16-20 weeks. The rabbits were anesthetized by intramuscular injection of Rompun (an anesthetic and muscle relaxant for animals; Bayer, Leverkusen, Germany) (50 mg/kg), and Ketalar (ketamine hydrochloride; Parke-Davis, Taipei, Taiwan) (50 mg/kg). Following infiltration with Xylocaine (1% lidocaine, Fujisawa, Osaka, Japan), a dorsal 6-cm midline incision (6 cm above and 1 cm below the posterior iliac crest) was made. This was followed by two paramedian fascial incisions (2 cm lateral to the midline). Following this, an intermuscular plane was easily developed in order to expose the transverse processes of L5 and L6 bilaterally. These transverse processes were decorticated using a rongeur. For the 12 animals from the study group, 1.0 × 10^8^ allogeneic MSCs (at passage 3) without differentiation loaded on a 4 × 1.5 × 0.3 cm^3^ sized scaffold were implanted on each side of the L5-6 intertransverse-process space. For the 12 rabbits from the control group, only the scaffold was implanted on each side of the L5-6 intertransverse-process space. The scaffold we used was a mixture consisting of bioresorbable purified fibrillar collagen and calcium phosphate ceramics containing hydroxyapatite (HA) and β-tricalcium phosphate (β-TCP) (Sunmax Biotechnology CO., Tainan, Taiwan). The highly purified collagen component was porcine dermal type I collagen. The ceramic portion was radiopaque and with a HA/β-TCP ratio of 65/35 - 60/40. All the animals were cared for in accordance with the regulations of the National Institutes of Health of the Republic of China (Taiwan), and this study protocol was approved by the Committee for Animal Experimentation of Chang Gung Memorial Hospital.

### Imaging analysis

**Figure 1:**
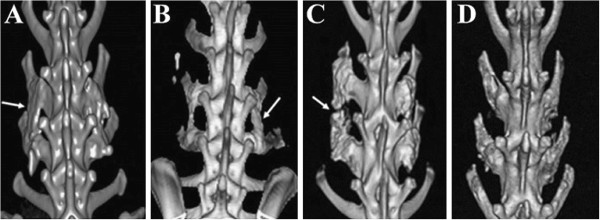
**Definition of fusion results. A**. Excellent: prominent osseous growth between bilateral transverse processes with remodeling (arrow). **B**. Good: moderate osseous growth with continuity between bilateral transverse processes (arrow). **C**. Fair: osseous growth between bilateral transverse processes with substantial clefts (arrow). **D**. Poor: scanty osseous growth between bilateral transverse processes.

All rabbits underwent 3-D CT scanning of the lumbar spine before they were sacrificed at 18 weeks. An excellent fusion was defined as prominent bilateral osseous growth between the transverse processes without a cleft and presence of remodeling on the fusion masses. A good fusion was defined as a bilateral moderate amount of osseous growth between the transverse processes without a cleft on the fusion masses. A fair fusion was defined as fusion with evidence of osseous growth, although substantial clefts are present between the transverse processes. A nonfusion was defined as scanty osseous growth with large clefts between the transverses processes (Figure 1). Excellent and good fusion was categorized as satisfactory fusion, while fair and poor fusion was categorized as unsatisfactory fusion. Data were analyzed using Fisher's exact test, and a *P* value of less than 0.05 was considered to represent a statistically significant difference between the study and control groups.

## Results

### Isolation and culture of rabbit bone marrow MSCs

**Figure 2:**
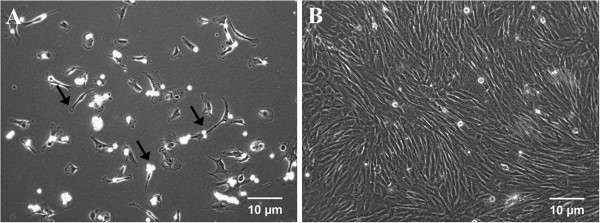
**Morphology of rabbit marrow cells under inverted microscope (original magnification, 100×). A**. Minimal fibroblast-like cells (arrows) adhering to the culture surface 1 week after seeding. **B**. A large amount of fibroblast-like cells adhering to the culture surface 2 weeks after seeding.

Under an inverted microscope, minimal fibroblast-like cells were found adhering to the culture surface at 1 week after plating (original magnification, 100×). However, a large amount of cells had adhered to the culture surface, and showed a fibroblast-like morphology at 2 weeks after plating (original magnification, 100×) (Figure 2). At 80-90% confluence, attached cells were harvested for subsequent studies.

### Differentiation potentials of cloned rabbit marrow cells

**Figure 3:**
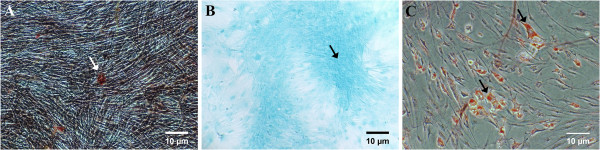
**Photomicrography (40×) of rabbit MSCs after differentiation and relevant staining. A**. Cells after osteogenic differentiation and staining showed calcium deposition (arrow). **B**. Cells after chondrogenic differentiation and staining showed glycosaminoglycans (GAGs) (arrow). **C**. Cells after adipogenic differentiation and staining showed accumulation of intracellular neutral lipid vacuoles (arrow).

**Figure 4:**
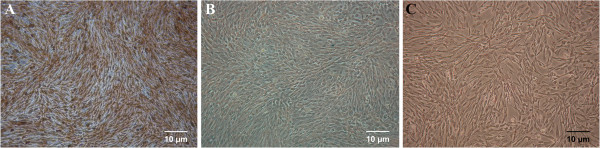
**Photomicrography (40×) of HIG-82 cells (rabbit fibroblasts) (control) after a series of staining. A**. Alizarin red staining showed no calcium deposition. **B**. Alcian blue staining showed no sulfated cartilage glycosaminoglycan. **C**. Oil Red O staining showed no intracellular accumulation of neutral lipid vacuoles.

The differentiation potentials of rabbit MSCs are shown in Figure 3. The cells after osteogenic differentiation and Alizarin red staining showed aggregates or nodules of calcium under an inverted microscope. Chondrogenic differentiation and Alcian blue staining showed an accumulation of sulfated cartilage glycosaminoglycans (GAGs), which is a component of articular cartilage. After adipogenic differentiation and Oil Red O staining, they showed intracellular accumulation of lipid-rich vacuoles. As for the control, the cloned HIG-82 cells (rabbit fibroblasts) that had undergone the same staining methods as described above showed no accumulation of calcium, sulfated cartilage glycosaminoglycan or neutral lipid (Figure 4). The differentiation test showed the cloned rabbit bone marrow cells had a differentiation capacity that was not present in fibroblasts (mature mesenchymal cells).

### Immunophenotypes of rabbit marrow cells

**Figure 5:**
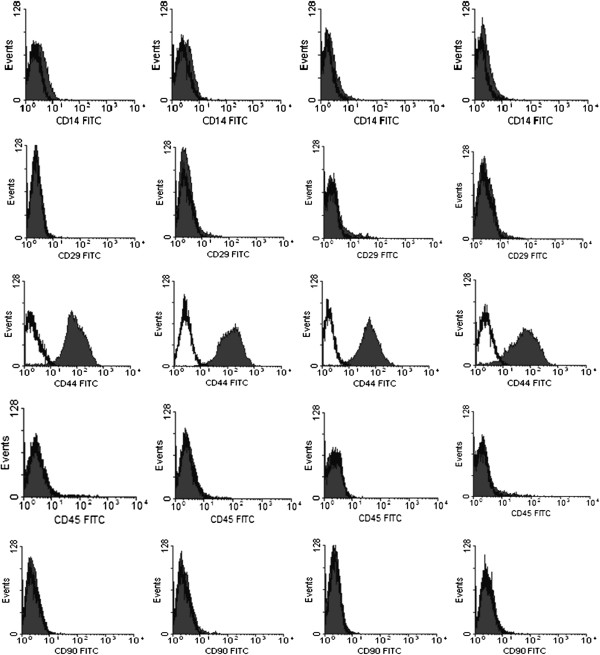
**FACS analysis (with 4 flow cytometry procedures) of our rabbit MSCs at passage 3.** The study showed that our rabbit MSCs were negative for CD14, CD29, CD45, and CD90 (column 1, 2, 4, 5), but positive for CD44 (column 3).

The rabbit MSCs at passage 3 cultured with anti-rabbit CD14, CD29, CD44, CD45, and CD90 antibodies displayed a stable phenotype (Figure 5). These antibody-labeled cells were analyzed 4 times with flow cytometry and the percentage of marker-positive cells is shown as Mean ± SD (n = 4) (Table 1). The current study showed that 2% of our cells expressed CD14, CD29 and CD45, and 1% expressed CD90. It also showed that 97% expressed CD44. Therefore, our rabbit MSCs were negative for CD14, CD29, CD45, and CD90, but positive for CD44.

**Table 1:** The digitalization of the FACS analysis of our rabbit MSCs at passage 3 showing percentage of marker-positive cells

	**1**	**2**	**3**	**4**	**Mean ± SD**
CD14	3.27	1.73	1.9	1.63	2.50 ± 0.77
CD29	0.67	4.28	2.78	3.09	2.71 ± 1.50
CD44	97	99.02	97	97	97.51 ± 1.01
CD45	4.5	0.38	2.78	2.77	2.61 ± 1.69
CD90	1	2.16	0.54	2.21	1.48 ± 0.84

### Imaging analysis

In the study group (n = 12), CT scanning revealed excellent fusion in 2 rabbits (17%), good fusion in 8 (66%), and fair fusion in 2 (17%). In the control-group (n = 12), a good fusion result was found in 3 rabbits (25%), fair fusion in 6 (50%), and poor fusion in 3 (25%). Excellent and good fusion was categorized as satisfactory fusion, while fair and poor fusion was unsatisfactory fusion.

On this basis, in the study group, satisfactory fusion was found in 10 rabbits (83%) and unsatisfactory fusion in 2 (17%). In the control group, satisfactory fusion was found in 3 rabbits (25%) and unsatisfactory fusion in 9 (75%). Statistical analysis using Fisher's exact test showed that the study group had significantly better bone fusion than the control group, with a *P* <0 .01.

## Discussion

Using Dominici’s definition [1], the immunophenotypic analysis in the current study showed our rabbit MSCs were negative for CD14, CD29, CD45, and CD90, while positive for CD44. However, positive reactivity for CD29 and CD90 was found on human MSCs [1,2,6,7,14,15]. Therefore, the surface markers of human and rabbit MSCs are not exactly the same. This finding has also been supported by other reports. Martínez-Lorenzo et al. found that more than 95% of human MSCs expressed CD90, but only 40% of rabbit MSCs at passage 1 expressed this marker [7]. Lapi et al. reported that CD90 was absent on rabbit MSCs, but they did not digitalize the percentage of rabbit MSCs that expressed CD90 [6]. To our knowledge, this is the first study to find that less than 2% (fulfilling Dominici’s definition of negative reactivity) of rabbit MSCs expressed CD90.

In the current study, CT analysis of the surgical results showed that rabbit MSCs promoted spinal fusion. The MSCs used in the current study were allogeneic undifferentiated cells. According to the literature, both autologous and allogeneic undifferentiated MSCs can heal bone defects in animals [16,17]. No immunosuppressive therapy was administered and no adverse immune response was detected in the model using allogeneic undifferentiated MSCs [17]. Actually, allogeneic undifferentiated MSCs could prolong allotransplant survival in a swine model by reducing the immune response [18]. Although some reports advocated using autologous MSCs with osteogenic differentiation to promote spinal fusion [19,20], we found this modality has shortcomings. First, the amount of harvested bone marrow cells is limited, so as not to endanger the animals. Second, it is time-consuming, as each animal has to wait for several weeks before the cloned autologous MSCs are available. In contrast, cryopreserved allogeneic MSCs isolated from any donor can provide a readily available source of cells for bone tissue engineering [17].

**Figure 6:**
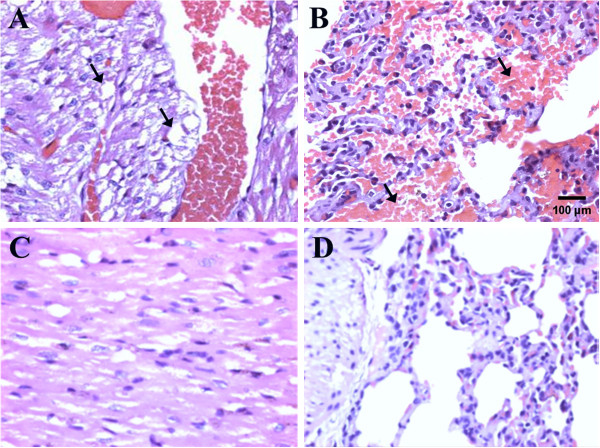
**Photomicrography (40×) of the rabbit organs.** In one rabbit that died after implantation of allogeneic differentiated MSCs, there was myocardial damage with vacuole formation (arrows) **(A)** and lung congestion (arrows) **(B)**. In one normal rabbit (control), the heart and lung showed no myocardial or pulmonary damage **(C,D)**.

We do not recommend using allogeneic differentiated MSCs to promote spinal fusion. Our preliminary data show that this modality resulted in an unacceptably high mortality rate for the animals (6/12, 50%). We hypothesized that this might be due to an adverse immune response. The postmortem organs of the rabbits that died within 3 days after implantation of allogeneic differentiated MSCs showed myocardial damage and pulmonary congestion (Figure 6).

Collagen has been reported to be useful as a scaffold for MSCs to repair bone or tendon defects [21,22]. Calcium phosphate ceramics, including hydroxyapatite (HA) and tricalcium phosphate (TCP), also have been introduced to carry MSCs to promote animal spinal fusion [23]. As such, many authors preferred using the combination of collagen and calcium phosphate ceramics as a scaffold for MSCs to facilitate spinal fusion [8,19]. The current study found scaffolds containing type I collagen and calcium phosphate ceramics (including HA and TCP) were effective as carriers for MSCs to promote fusion.

Our results demonstrated the variation of immunophenotypes among human and rabbit bone marrow mesenchymal stem cells. In addition, local implantation of allogenic undifferentiated stem cells could possibly be applied in promoting spinal fusion in the future.

## Conclusions

Immunophenotypic analysis in the current study showed the surface markers of human and rabbit MSCs are not exactly the same. The rabbit MSCs showed negative for CD29 and CD90, while the human MSCs invariably showed positive for these two markers. The fusion results of the current study showed that allogeneic undifferentiated rabbit MSCs did not induce an adverse immune response and could enhance spinal fusion.

## Abbreviations

DMEM-LG: Low-glucose Dulbecco's Modified Eagle Medium; FACS: Fluorescence activated cell sorter; FBS: Fetal bovine serum; FITC: Fluorescein isothiocyanate; HA: Hydroxyapatite; MSCs: Mesenchymal stem cells; PBS: Phosphate-buffered saline; TCP: Tricalcium phosphate.

## Competing interests

The authors declare that they have no competing interests.

## Authors’ contributions

THL and YHH led all aspects of this study, including experimental design, data analysis and interpretation. TCL conceived of the study, carried out all procedures involving rabbits, and drafted the manuscript. NKC, WCL, PWCC, and TMS participated in the preparation of the study materials. DJH made substantial contributions to the provision of study materials. All authors read and approved the final manuscript.
